# Injectable Bulking Agent to Treat Postprostatectomy Urinary Incontinence: A Safety and Effectiveness Pilot Study

**DOI:** 10.1155/2018/2796967

**Published:** 2018-12-06

**Authors:** Janneke I. M. van Uhm, Marloes Vermeer, Henk W. Elzevier, Joop W. Noordzij, Evert L. Koldewijn, Erik B. Cornel

**Affiliations:** ^1^Department of Urology, Leiden University Medical Center, 2 Albinusdreef, P.O. Box 9600, Leiden 2300 RC, Netherlands; ^2^Ziekenhuisgroep Twente, 141 Geerdinksweg, P.O. Box 546, Hengelo 7550 AM, Netherlands; ^3^Amstelland Ziekenhuis, 8 Laan van de Helende Meesters, P.O. Box 328, Amstelveen 1180 AH, Netherlands; ^4^Catharina Ziekenhuis, 2 Michelangelolaan, P.O. Box 1350, Eindhoven 5602 ZA, Netherlands

## Abstract

**Objectives:**

To evaluate the safety and effectiveness of the injectable bulking agent Opsys® (Promedon, Cordoba, Argentina) for treating minimal postprostatectomy stress urinary incontinence (SUI).

**Patients and Methods:**

Single-centre, pilot study on ten male patients with SUI, < 30 g urine loss/ 24 h, more than 1 year after radical prostatectomy. Patients were treated by endoscopic transurethral injections of bulking agent in the presphincteric zone of the urethral submucosa. The results were evaluated using a pad weight test to quantify the differences in urine loss at 1, 3, and 6 months after intervention. Subsequently, the results of treatment were also evaluated by International Consultation on Incontinence Questionnaire Short Form (ICIQ-SF), Incontinence Impact Questionnaire (IIQ-7), Urogenital Distress Inventory Short Form (UDI-6-SF), and the Patient Global Impression of Improvement (PGI-I) at 1, 3, and 6 months after intervention.

**Results:**

The primary outcome was the absolute result of the 24-hour pad weight test after treatment. Treatment success was defined as <3 g urine loss/24 h, improvement as ≥50% decrease in urine loss/ 24h, failure as <50% decrease in urine loss/24 h, or worsening of urine loss. Success was demonstrated in one, improvement in one, and failure in eight patients one month after treatment. One patient improved and 9 failed 3 and 6 months after treatment. The median 24-hour pad weight test was higher at all three moments of follow-up (1, 3, and 6 months after treatment). The median 24-hour pad weight test was before treatment 17.3g (6.4-20.9) and 1, 3, and 6 months after treatment, respectively, 40.3g (5.9-130.6) p= 0.038, 38.3g (18.3-202.1) p= 0.014, 55.0g (16.5-314.6) p= 0.028. The ICIQ-SF was significantly higher at 3 and 6 months, respectively 15.0 (12.0-18.5) p= 0.007 and 16.0 (12.5-17.5) p=0.012 versus 10.0 (9.0-12.0) before injection. No significant differences were found between IIQ-7, UDI-6-SF, and PGI-I before and after injection. Complications occurred in four patients: two patients reported spontaneously resolved haematuria and two patients reported urinary frequency. All complications were classified as Clavien–Dindo 1.

**Conclusion:**

Injection therapy with Opsys® bulking agent is not an effective treatment option for male SUI after radical prostatectomy. It is not a safe treatment option, due to worsening urine loss after treatment.

## 1. Introduction

Urinary incontinence after radical prostatectomy has a high impact on patients' quality of life. The current guidelines on postprostatectomy urinary incontinence stated that surgical treatments can be considered for men who fail conservative treatment [1]. The artificial urinary sphincter is still considered the gold standard for treating postprostatectomy incontinence [2]. The male sling and Proact balloons, however, have proved to be good alternative treatment options for patients with mild postprostatectomy incontinence [3]. However, there is still no appropriate minimal invasive intervention available for minimal postprostatectomy incontinence (<30 g urine loss/24-hour pad test), though injection of a bulking agent might be a solution. Multiple bulking agents have been used for treatment of female stress urinary incontinence (SUI) with inconsistent results on effectiveness [4]. The Opsys® (Promedon, Cordoba, Argentina) bulking agent seemed to be safe and effective for treating female SUI and can be offered as a minimally invasive procedure with quite durable clinical results and minimal complications [5]. Opsys® is made of a polyacrylate polyalcohol copolymer, and it is a nonabsorbable biomaterial which is also used in children to treat vesicoureteral reflux [6]. The biocompatibility and nonmigration characteristics as well as long term bulking stability in the injection site of Opsys® have been studied in in-vivo and in-vitro studies [7]. Various kind of bulking agents have been used to treat male incontinence, the outcome was variable, and the reintervention rate of 52.9% was high [8]. However, there are no scientific data regarding the efficacy and safety of Opsys® for treating postprostatectomy incontinence [8]. We hypothesised that injection of the bulking agent Opsys® might be an appropriate minimal invasive treatment for patients with minimal SUI after prostatectomy. The expectation was that bulking agents could replace the more invasive and more comorbidity-related implantation of slings and Proact balloons for these patients. We present a pilot study that evaluated the safety and efficacy of the injectable bulking agent Opsys® for treating postprostatectomy SUI.

## 2. Materials and Methods

### 2.1. Inclusion and Exclusion Criteria

All patients gave their written informed consent before inclusion in the study. The study was approved by the local ethics committee and registered in the Dutch Trial registration as number NL.57054.044.15. Patients were included in this pilot study and treated with Opsys® bulking agent between October and December 2016. Inclusion criteria were minimal SUI (<30 g/day loss during the 24-hour pad weight test), at least 12 months after radical prostatectomy, and being refractory to conservative treatment, such as pelvic floor muscle training. Patients remained dry at night and could voluntarily stop micturition. Patients with a history of radiation treatment for prostate carcinoma, bladder neck sclerosis, urethral stricture, urgency urinary incontinence, detrusor overactivity during urodynamic evaluation, and/or urinary tract infection were excluded from the study.

### 2.2. Bulking Agent

Opsys® consists of particles of polyacrylate polyalcohol copolymer which is a nonabsorbable biomaterial. It has a very high molecular mass (~10,000 kDa) and comes in the form of sterile pyrogen-free particles. The macroparticles have an average diameter of 300 *μ*m and the carrier is a 40% glycerol solution. This substance can be manually injected easily through small needles (21-gauge). Once implanted, the glycerol solution is eliminated by the reticuloendothelial system without metabolizing it and is excreted through the kidneys, leaving the particles behind for permanent bulking [7].

### 2.3. Injection Procedure

Broad-spectrum antibiotics were administered. General or regional anaesthesia was given based on patients' preference. All procedures were performed in the operating room. Opsys® was implanted using a video endoscope with 6-French working channel, 0° optics, and a 21-gauge transurethral injection needle. All procedures were video-recorded. The bulking agent was injected in a transurethral manner into the presphincter zone of the urethral submucosa. Injections of about 1.0 mL of bulking agent around all quadrants in the urethra were executed. The bladder was emptied after the procedure by a single use catheter 12 French.

### 2.4. Follow-Up

All patients were assessed prior to treatment via their medical history, physical examination, and endoscopic- and urodynamic evaluations including postvoid residual volume. All patients were assessed again 1, 3, and 6 months after treatment. Pretreatment and posttreatment SUI were evaluated using two 24-hour pad weight tests. Posttreatment success was defined as a maximum of 3 g of urine loss during the 24-hour pad weight test. Improvement was defined as a ≥50% reduction of urine loss during the 24-hour pad weight test. Failure was classified as <50% reduction or increased urine loss during the 24-hour pad weight test.

Uroflowmetry and postvoid residual volume was measured each post treatment visit.

The following validated questionnaires were administered preoperatively and at every follow-up visit: International Consultation on Incontinence Questionnaire Short Form (ICIQSF), Incontinence Impact Questionnaire (IIQ-7), and Urogenital Distress Inventory Short Form (UDI-6-SF). In addition, the Patient Global Impression of Improvement (PGI-I) was assessed postoperatively [1]. Complications were assessed and classified using the Clavien–Dindo classification [9].

### 2.5. Statistical Analysis

#### 2.5.1. Power Calculation

A total of 30 subjects were planned to be included in this clinical study, distributed among two research centres. The inclusion of 30 patients within the given time period was feasible for the two centres. Injection of Opsys® was expected to result in a success percentage of 30-50%. The sample size calculation was based on the two-sided confidence interval formula (Score (Wilson)) for one proportion. This was calculated using PASS 11. A sample size of 30 produces a two-sided 95% confidence interval with a width equal to 0.337 when the sample proportion is 0.500: 0.332 to 0.668.

#### 2.5.2. Follow-Up Data

Continuous normally distributed variables are reported as means with standard deviation (SD), and continuous nonnormally distributed variables are presented as medians with interquartile range (IQR). The preoperative and postoperative (1, 3, and 6 months) 24-hour pad weight test ICIQ-SF, IIQ-7, UDI-6-SF, and PGI-I scores were compared using the Wilcoxon signed-rank test. To analyse the PGI-I score at 1, 3, and 6 months, we adapted a baseline value of 4.0, which indicated no change.

A p-value <0.05 was considered to be statistically significant. All data were analysed using SPSS version 23.0.

## 3. Results

Ten patients were included in the pilot study. The calculated power of 30 patient was not accomplished due to an inclusion stop after interim analysis of the effectiveness and safety results of the first 10 patients.  Table 1 summarizes the baseline characteristics. The mean patient age was 67.0 years (range 55–77 years). The mean BMI was 29.7 kg/m^2^ (range 21-44 kg/m^2^). The following comorbidities were present: hypertension (n=4), diabetes mellitus (n=3), hypercholesterolemia (n=2), sleep apnoea syndrome (n=1), and chronic obstructive pulmonary disease (n=1). Three patients did not have significant comorbidities. The postvoid residual volume was in all patients < 50 mL.

The mean number of transurethral injections per patient was 4.7 (range 3–7). The mean total injected volume of bulking agent was 2.3 mL (0.3–5.0 mL).

Complications occurred in four patients. Two patients had light haematuria for 2–4 days after the injection that resolved by itself. Two patients reported urinary frequency, one patient for 1 day after the injection and one patient for 2 months. All complications were classified as Clavien–Dindo 1.

The results after 1 month showed one patient with success, one with improvement, and eight with failure (Table 2). At 3 and 6 months' follow-up, none of the patients showed a successful outcome: one patient exhibited improvement and nine patients showed failure. The median 24-hour pad weight test was significantly higher 1, 3, and 6 months after injection of the bulking agent, respectively, 40.3 g (5.9-130.6) p= 0.038, and 38.3 g (18.3-202.1) p= 0.014, 55.0 g (16.5-314.6) p= 0.028, compared with baseline, 17.3 g (6.4-20.9). Posttreatment clinical examination/interview demonstrated no signs of urge incontinence. Posttreatment residual volume was in all patients below 50 mL.

The ICIQ-SF was significantly higher after 3 months, 15.0 (12.0-18.5) p= 0.007, and 6 months, 16.0 (12.5-17.5) p=0.012, compared with baseline, 10.0 (9.0-12.0) (Table 2). No significant differences were found between the IIQ-7, UDI-6-SF, and PGI-I scores before and after injection.

## 4. Discussion

SUI greatly affects patients' quality of life. The minimally invasive procedure that entails injecting the bulking agent Opsys® in patients who have minimal SUI failed in 9 of the 10 patients. Consequently, the inclusion of patients in the study was stopped before the calculated power of 30 patients was reached. Furthermore, the follow-up in the study was shortened by 6 months instead of 12 months in the initial protocol. So, patients were able to get another treatment for incontinence. Subsequently, one patient was treated with an artificial urinary sphincter and two patients with a male sling. All three patients were dry after this reintervention. The remaining 6 patients who did not improve after injection treatment refused an additional treatment. A major weakness of this study is the small sample size and the lack of an appropriate control group. However, it is also important to publish these results and to suggest explanations for the poor outcome of the injection of the bulking agent Opsys®.

First, three independent urologists expert in male urinary incontinence evaluated the recorded videos of the endoscopic injection procedures of the patients. They provided a blinded prediction of the functional outcome of each injection procedure. There was no correlation between any of the predicted results and the real-time outcomes. The experts concluded that all injection procedures were performed according to the technical description in the protocol. The independent urologist checked for the following eight steps of injection procedures: (1) The endoscope was introduced in the bladder and the injection needle in the endoscope till the tip of the needle was seen; (2) the endoscope and the tip of the needle removed backward up to the presphincter zone; (3) the needle was injected at a 30°-45° angle with regard to the urethral mucosa; (4) the endoscope was placed at a 0° angle, parallel to the urethra; (5) the position of the needle was checked injecting a small amount of product, which demonstrated bulkiness in the urethral submucosa and around 1 mL Opsys® was injected; (6) the needle was removed from the injection site after 15-30 seconds; (7) the clinician injected at four quadrants in the urethral submucosa or till bulkiness and closing of the urethral lumen was observed; (8) the endoscope was not moved forward through the injection site, since this could deform injected material bulkiness.

**Figure 1:**
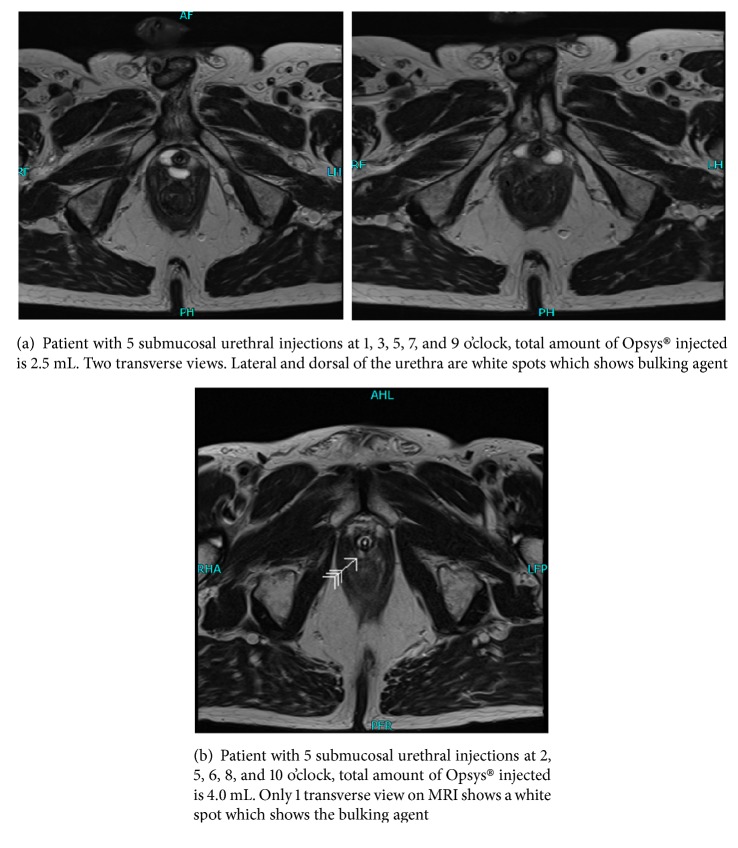
**Magnetic resonance imaging (MRI** **∗** **) views of bulking agent**. *∗1.5 T Siemens Avanto MRI:T2 Blade 3,5mm sagittal, T1 space coronal 0.9mm, T2 TSE transversal 3.5mm, T2 TSE coronal oblique 3.5mm, and T2 TSE coronal 3.5mm. Technical aspects: Field Of View 240mm, distance 30%, phase right to left, resolution 320, and phase resolution 86%.
*

Second, we used magnetic resonance imaging (MRI) of the pelvis/urethra to evaluate the anatomical positioning of the bulking agent after injection. Postinjection MRI was not part of the protocol. However, we performed postinjection MRI in all patients to investigate the poor outcome after injection. A radiologist of the study centre and two authors of this manuscript assessed the MRI results. It showed no correlation between the demonstrated MRI bulking agent around the urethra and the number of injections, volume of the injections, location of injections, or functional outcome (Figure 1). The differences between location/volume of injection and the MRI results are difficult to explain. There should be no risk of migration around the injection site or to other parts of the body because of the 300 *μ*m average diameter of the polyacrylate polyalcohol copolymer particles. Because these macroparticles are flexible, irregularly shaped, and highly deformable by compression, they may be extruded using 21-gauge needles. Once implanted, macroparticles enlarge the volume of the tissue, generating little fibrotic growth around them (i.e., 70–125 *μ*m thick) [7]. Subsequently, MRI evaluation of the injection technique and the anatomical position of the bulking agent did not result in an explanation of failure or even worsening of the urinary incontinence. The results of our study were therefore disappointing compared with the former results of Opsys® in women with SUI [5]. Hence, we must address the causes of the failed treatment of male SUI after radical prostatectomy.

Figures 1(a) and 1(b) MRI views demonstrated different location and amount of bulking agent on 3.5 mm slides. Both patients had 5 injections with bulking agent. The urethra can be recognized by the transurethral catheter.

Postprostatectomy urinary incontinence is due to damage of the anatomic support and pelvic innervation. The anatomic support consists of the urethral sphincter complex for passive and active continence and the anterior and posterior support structures around the urethra and the pelvic floor [10]. Women also have internal sphincter deficiency, but the aetiology of urinary incontinence is different between the sexes. In men, pelvic surgery leading to neurovascular and anatomic support damage is the main cause, while birth-related trauma is the main cause in women. The trauma that induces urinary incontinence in men tends to occur at an older age (sixties onwards) than in women (childbearing age). The collagen tissue surrounding the urethra might be less supportive in older patients, especially if there is neurovascular damage. The bulking agent around the urethra might have further diminished the vascularisation of the urethra due to obstruction by the bulk material. Our results compare unfavourably to earlier results with Opsys® in the treatment of female SUI and other published results on bulking agents in the treatment of male SUI. One patient showed increased incontinence (>1000mL / 24 hours) and was additionally treated by an artificial urinary sphincter implant. To our knowledge, this worsening of incontinence after bulking agent injection was never published. The former published studies on transurethral injection of collagen demonstrated a short-term success rate of 44–58%. None of those studies, however, were randomised or placebo-controlled. A subsequent review published in 1996 concluded that transurethral injection with collagen has a limited role in the management of urinary incontinence [11]. Macroplastique injections demonstrated a success rate of 43% and even dry rates up to 80% in a selected group of patients with minimal incontinence [12, 13]. Chughtai et al. evaluated all interventions for SUI or mixed incontinence in male patients during 2000–2011 [8]. This study showed that the use of bulking agent has decreased from 52.2% to 16.4% in favour of sling surgery, which increased from 14.8% to 51.4%. One of the reasons for this might be the higher reintervention rate during the first year following surgery in patients treated with bulking agents (40.1%) compared with the sling (9.7%) and an artificial urinary sphincter (7.1%). Our hypothesis regarding the failure of Opsys® bulk injection in the present study is that postprostatectomy urinary incontinence has multifactorial causes, including anatomic support and pelvic innervation damage. These multifactorial causes are not resolved by injecting this bulking agent.

## 5. Conclusions

Injection of Opsys® bulking agent is not a safe and effective treatment option for male SUI after radical prostatectomy. The treatment resulted in worsening of the minimal SUI. We could not find an evident explanation for these results. So, we could not suggest adaptions to improve the treatment with Opsys® bulking agent in male SUI.

## Figures and Tables

**Table 1:** Baseline characteristics.

**Variable**	**N=10**
Age (years), mean ± SD	67.0 ± 6.1
BMI (kg/m^2^), mean ± SD	29.7 ± 6.3
Mode of previous prostate surgery, n (%)	
Open radical prostatectomy	5 (50)
Robot-assisted radical prostatectomy	4 (40)
Laparoscopic radical prostatectomy	1 (10)
Anaesthesia, n (%)	
Spinal	5 (50)
General	5 (50)
Operation time (min), mean ± SD	14.1 ± 3.8
Number of injections, mean ± SD	4.7 ± 1.1
Total injected volume (mL), mean ± SD	2.3 ± 1.5

**Table 2:** Postoperative results. P-values correspond to the Wilcoxon signed-rank test for comparing baseline with 1, 3 and 6 months follow-up. ICIQ-SF = International Consultation on Incontinence Short Form; IIQ-7 = Incontinence Impact Questionnaire; UDI-6-SF = Urogenital Distress Inventory Short Form; PGI-I = Patient Global Impression of Improvement. *∗∗* n.c. = no change; for statistical analysis we adopted a baseline value of 4.

	**Baseline**	**1 month**	***P***	**3 months **	***P***	**6 months**	***P***
		**(n=10)**		**(n=10)**		**(n=10)**	
Treatment outcome, n (%)							
Success		1 (10)		0 (0)		0 (0)	
Improvement		1 (10)		1 (10)		1 (10)	
Failure		8 (80)		9 (90)		9 (90)	
24-h pad weight test (g), median (IQR)	17.3 (6.4 – 20.9)	40.3 (5.9 – 130.6)	0.038	38.3 (18.3 – 202.1)	0.014	55.0 (16.5 – 314.6)	0.028
ICIQ-SF score, median (IQR)	10.0 (9.0 – 12.0)	16.0 (11.8 – 18.0)	0.109	15.0 (12.0 -18.5)	0.007	16.0 (12.5 – 17.5)	0.012
IIQ-7 score, median (IQR)	26.5 (13.0 – 41.5)	38.0 (34.5 – 50.3)	0.122	49.5 (17.8 – 67.0)	0.413	36.0 (15.5 – 62.0)	0.528
UDI-6-SF score, median (IQR)	33.0 (20.8 – 40.3)	36.0 (26.5 – 44.0)	0.553	39.0 (17.0 – 58.5)	0.552	39.0 (28.0 – 47.0)	0.766
PGI-I score, median (IQR)	n.c.*∗∗*	5.5 (4.8 – 6.0)	0.102	4.5 (3.8 – 6.0)	0.121	5.0 (3.5 – 5.0)	0.206

## Data Availability

The data used to support the findings of this study are available from the corresponding author upon request.
